# Dual mechanisms in hydrogen reduction of copper oxide: surface reaction and subsurface oxygen atom transfer

**DOI:** 10.1039/d4ra01240b

**Published:** 2024-03-26

**Authors:** Yehan Wu, Ruixue Fang, Laihong Shen, Hongcun Bai

**Affiliations:** a Key Laboratory of Energy Thermal Conversion and Control of Ministry of Education, School of Energy and Environment, Southeast University Nanjing 210096 China; b State Key Laboratory of High-efficiency Utilization of Coal and Green Chemical Engineering, Ningxia University Yinchuan Ningxia 750021 China

## Abstract

The study of the reduction of copper oxide (CuO) by hydrogen (H_2_) is helpful in elucidating the reduction mechanism of oxygen carriers. In this study, the reduction mechanism of CuO by H_2_ and the process of oxygen atom transfer were investigated through the density functional theory (DFT) method and thermodynamic calculation. DFT calculation results showed that during the reaction between H_2_ and the surface of CuO, Cu underwent a Cu^2+^ → Cu^1+^ → Cu^0^ transformation, the Cu–O bond (−IpCOHP = 2.41) of the Cu_2_O phase was more stable than that (−IpCOHP = 1.94) of the CuO phase, and the reduction of Cu_2_O by H_2_ was more difficult than the reduction of CuO. As the surface oxygen vacancy concentration increased, it was more likely that the subsurface O atoms transfer to the surface at zero H_2_ coverage (no H_2_ molecule on the surface), allowing the surface to maintain a stable Cu_2_O phase. However, when the H_2_ coverage was 0.25 monolayer (ML) (one H_2_ molecule every four surface Cu atoms), the presence of H atoms on the surface made the upward transfer of O atoms from the subsurface more difficult. The rate of consuming surface O atoms in the reduction reaction was greater than the rate of subsurface O atom transfer induced by the reduction reaction and the surface Cu_2_O phase could not be maintained stably. Through thermodynamic analysis, at high H_2_ concentration, the reaction between H_2_ and CuO was more likely to generate Cu, while at low H_2_ concentration, it was more likely to generate Cu_2_O. In summary, the valence state of Cu in the reaction process between CuO and H_2_ depended on the concentration of H_2_.

## Abstract 2

The surface reaction and subsurface oxygen transfer jointly affect the process of hydrogen reduction of copper oxide.

## Graphical abstract

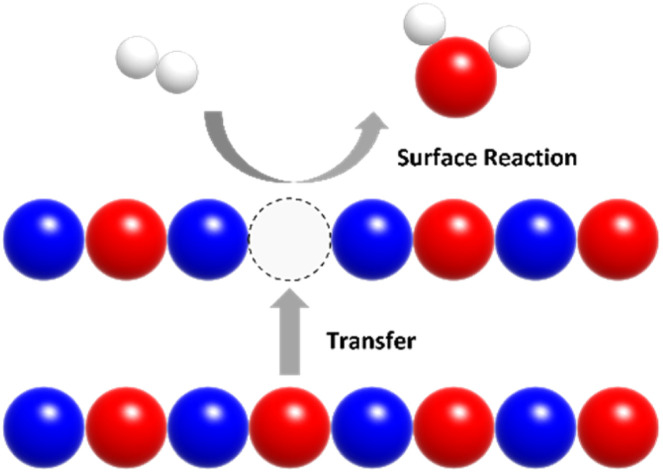

## 1 Introduction

Chemical looping combustion (CLC) has been shown to be a promising combustion technology for power production with integrated CO_2_ capture.^1–3^ In CLC, a metal oxide that acts as an oxygen carrier is alternately reduced by a fuel (syngas or natural gas) in a fuel reactor and oxidized by air in an air reactor. CuO is a good oxygen carrier for chemical looping combustion.^4–6^ CuO undergoes a reduction process in the fuel reactor. Numerous studies have been carried out on CuO. However, the reduction mechanism of CuO, especially the influence of reducing gas concentration, is currently unclear. Hydrogen (H_2_) is the most common reducing gas. There were many studies on the reduction of CuO by H_2_. The reaction of H_2_ with CuO was experimentally studied using *in situ* X-ray diffraction by Kim *et al.*^7^ Samples were loaded in an open sapphire capillary having an inner diameter of 0.5 mm. An inlet gas was a 5% H_2_ and 95% He gas mixture. The results showed that under high H_2_ flow rate (≈15 cm^3^ min^−1^), CuO can be directly reduced to metallic Cu (Cu^2+^ → Cu^0^). During the reduction process, no evidence of any intermediate state (Cu^+^) was found. Cu_2_O appeared as an intermediate phase only at very low H_2_ flow rates (<1 cm^3^ min^−1^); this result has been supported by multiple studies.^8–11^

Maimaiti *et al.*^12^ investigated the process of H_2_ reduction on the surface of CuO using the density functional theory (DFT) method and concluded that Cu^+^ appeared during the reaction. This could not explain the experimentally observed phenomenon that Cu^2+^ is directly converted into the Cu^0^ phase under high H_2_ concentration. This may be due to the fact that, in addition to the reaction between H_2_ and the CuO surface, other reaction processes are involved in the reduction reaction. Using the DFT method, Jabraoui *et al.*^13^ found that oxygen vacancies play an important role in the decomposition of CuO to release O_2_. Oxygen vacancies on the surface can promote the transfer of internal O to the surface. Yang *et al.*^14^ found that rearrangements occurred on the CuO surface as the concentration of surface oxygen vacancies increased. Transition in the surface structure caused by the reaction between H_2_ and CuO may in turn affect the reaction between H_2_ and CuO.

In this study, the DFT method was adapted to study the reaction between H_2_ and the CuO surface initially. Subsequently, subsurface O atom transfer was studied with different H_2_ coverages (0 ML, 0.25 ML) by DFT. DFT provided a precise approach^15,16^ to the theoretical problem of electronic structure. Both the climbing image nudged elastic band (CI-NEB)^17^ and dimer^18^ methods were applied to find the transition states. The transition state structure of chemical reactions could help us better understand the kinetic properties of chemical reactions. Thermodynamic calculations were applied to the reactions occurring in the H_2_ reduction of CuO process.

## 2 Methodology and model

### 2.1 Computational methodology

All DFT calculations were performed using the software Vienna *ab initio* simulation package (VASP).^19,20^ The projector augmented wave (PAW) method was chosen for the calculations to handle the effect of the core electrons on the valence electrons.^21^ The Perdew–Burke–Ernzerhof (PBE) exchange function under the generalized gradient approximation (GGA) was selected.^22^ The Monkhorst–Pack method was used to sample the Brillouin zone,^23^ and *k*-point grids with sizes of 8 × 8 × 8 and 4 × 4 × 1 were used for CuO unit cells and CuO slab calculations, respectively. The plane wave cutoff energy was 400 eV. Because the conventional DFT functionals cannot accurately describe the strong correlation effect between the Cu 3d orbital electrons in CuO,^24^ the GGA+*U* method^25^ was used in order to accurately describe the strongly correlated, highly localized 3d electrons in CuO. To describe the Cu atom, *U* = 7 eV and *J* = 0.98 eV were used.^26^ The electron energy was relaxed to an accuracy of 10^−6^ eV, and the atomic positions were optimized until the Hellmann–Feynman force was less than 0.02 eV Å^−1^. Projected crystal orbital Hamilton population (pCOHP) calculations were conducted using the LOBSTER code.^27^ The structures were visualized by VESTA software.^28^ Thermodynamic calculations were conducted using the HSC Chemistry 6.0 software. For H_2_ adsorption on the CuO surface, the energy of gas adsorption is

1 *E*_ads_ = *E*(H_2_-surface) − *E*(surface) − *E*(H_2_)

where *E*_ads_ is the gas adsorption energy, *E*(H_2_-surface) is the total energy of the system after H_2_ is adsorbed to the CuO surface, *E*(surface) is the total energy of the CuO surface, and *E*(H_2_) is the total energy of H_2_. The activation energy of the reaction is expressed as

2 *E*_a_ = *E*_TS_ − *E*_IS_

where *E*_TS_ is the energy of the transition state and *E*_IS_ is the energy of the initial state. The heat of reaction is

3 Δ*E* = *E*_FS_ − *E*_IS_

where *E*_FS_ is energy of the final state. Microscopically, gas concentration can be reflected by the adsorption surface gas coverage (*θ*).^29^ The formula for adsorption gas coverage is as follows:

4 *θ* = *N*_g_/*N*_M_

where *N*_g_ is the number of adsorbed gas molecules, and *N*_M_ is the number of surface metal atoms. The unit for *θ* is monolayer (ML).

### 2.2 Structural properties

**Fig. 1:**
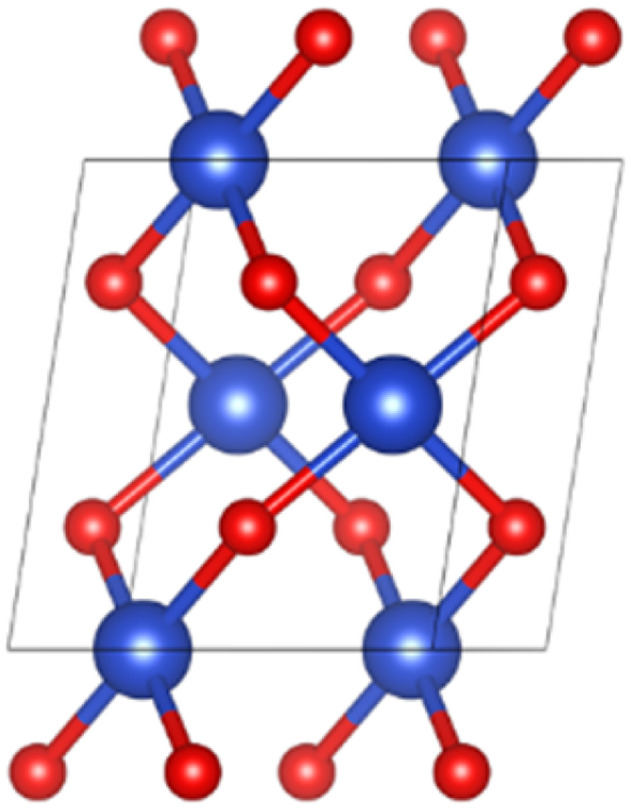
Unit cell structure of CuO.

The spatial structure of CuO crystal belonged to the *C*2/*c* group.^30^Fig. 1 illustrates the unit cell structure of CuO. The CuO unit cell parameters were calculated as *a* = 4.63 Å, *b* = 3.43 Å, *c* = 5.09 Å, *β* = 99.43°, and the Cu–O bond length was 1.95 Å, which was well aligned with experimental data,^30^ proving that the chosen parameter settings were appropriate.

**Fig. 2:**
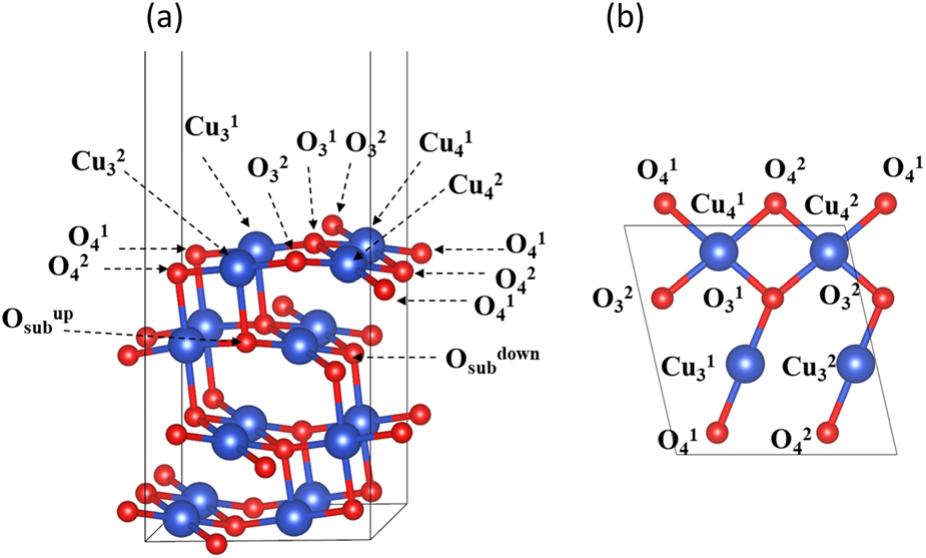
CuO (111) surface unit cell: (a) front view (b) top view.

Experiments and calculations have both demonstrated that CuO (111) is the most stable surface of copper oxide and the surface most easily observed in experiments.^31,32^ Therefore, the CuO surface model used in this study was CuO (111), as shown in Fig. 2. The CuO (111) surface used for hydrogen reduction and surface structure calculation was a (2 × 1) supercell that retains four layers of atoms. The thickness of the vacuum layer was 15 Å. The lower two of these layers were fixed, and the upper two were relaxed. Inside the unit cell, there were a total of four oxygen atoms on the surface of CuO (111), which were divided into two categories according to the coordination number. The oxygen atoms with a coordination number of three were labeled O^1^_3_ and O^2^_3_, and those with a coordination number of four were labeled O^1^_4_ and O^2^_4_. There were four Cu atoms on the surface in total, namely, the three-coordinated Cu^1^_3_ and Cu^2^_3_ and the four-coordinated Cu^1^_4_ and Cu^2^_4_.

## 3 Results and discussion

### 3.1 Study of the complete reaction paths of hydrogen on copper oxide surface

On the surface of intact copper oxide, two H_2_ adsorption sites were considered: the top sites of the three-coordinated Cu and four-coordinated Cu atoms. On the intact CuO surface, the adsorption energy *E*_ads_ of H_2_ on Cu^1^_3_ was −2.30 kJ mol^−1^, and *E*_ads_ of H_2_ on Cu^1^_4_ was −2.01 kJ mol^−1^. H_2_ adsorption on the intact surface of CuO released very little heat, indicating that no chemisorption occurred. However, H_2_ adsorption on the three-coordinated Cu^1^_3_ still released more energy, and it could be assumed that H_2_ was initially adsorbed on the three-coordinated Cu^1^_3_.

Next, the type of O that H_2_ reacts with first on the surface was considered. The reaction heat Δ*E* between H_2_ and O^1^_3_ was −182.50 kJ mol^−1^ and that between H_2_ and O^1^_4_ was −93.31 kJ mol^−1^.

H_2_ was more likely to combine with the three-coordinated O atoms on the surface of CuO to form H_2_O, so H_2_ would react with the three-coordinated O on the surface of CuO first, and then with the four-coordinated O afterward. This has also been confirmed by calculations by Maimaiti *et al.*^12^

**Fig. 3:**
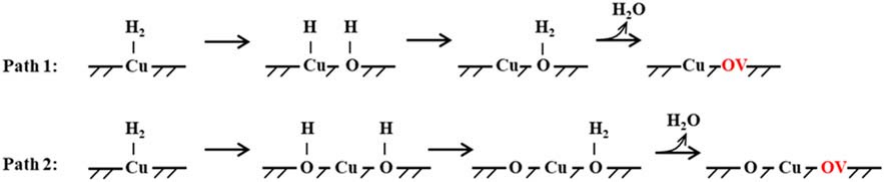
Two H_2_ dissociation modes and CuO reaction paths.

**Fig. 4:**
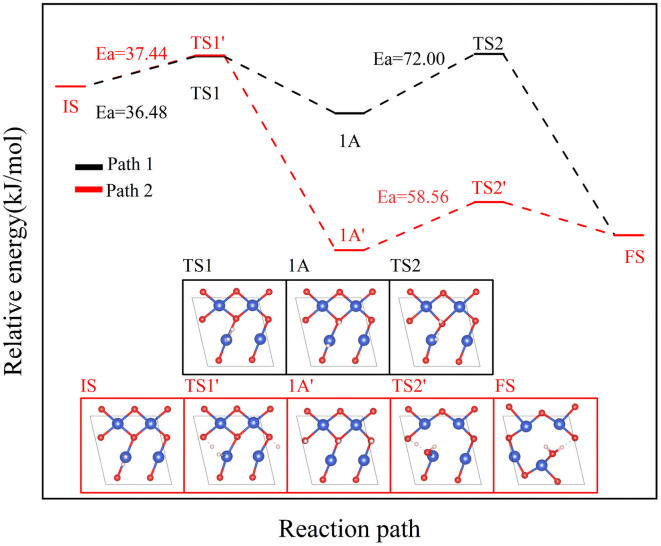
The two reaction paths of H_2_ with CuO (the white atom is the H atom).

The reaction of H_2_ with the CuO surface is divided into two parts. First, the dissociation of H_2_, and then the formation of H_2_O from the two dissociated H atoms and a surface O atom. H_2_ dissociates in two ways on the surface of metal oxides:^33–36^ the first is the dissociation of H_2_ to form H^−^ and H^+^, which are adsorbed on the metal and oxygen sites, respectively, producing M–H and O–H groups (path 1); the other is the dissociation of H_2_ to produce two H atoms, which combine with two O atoms to form two O–H groups (path 2). Both H_2_ dissociation pathways are possible during the H_2_ reduction of CuO. The two reaction paths of H_2_ with CuO are shown in Fig. 3. The energy changes in the process for the two different reactions paths are shown in Fig. 4. In path 1, from 1A to FS was the rate-determining step. In path 2, from 1A′ to FS was the rate-determining step. In path 2, the rate-determining step was an endothermic reaction, which increased the difficulty of the reaction. *X*_a_ was used to measure the similarity between the initial and final states, *i.e.*, the root sum squared of the distance between the initial and final states of the corresponding atoms. The smaller *X*_a_ was, the smaller the distance of the atom movement before and after the reaction was. In path 1, the *X*_a_ between 1A and IS was 2.45, and the *X*_a_ between FS and 1A was 3.68. In path 2, the *X*_a_ between 1A′ and IS was 3.89, and the *X*_a_ between FS and 1A′ was 4.09. From the perspective of H atom diffusion, in path 2, H atoms required more diffusion movement, which increased the difficulty of the reaction in path 2. Hence, the H_2_ dissociation approach chosen in this study is path 1.

Next, the full-path process of H_2_ reduction in the surface reaction of CuO was studied by DFT method.

**Fig. 5:**
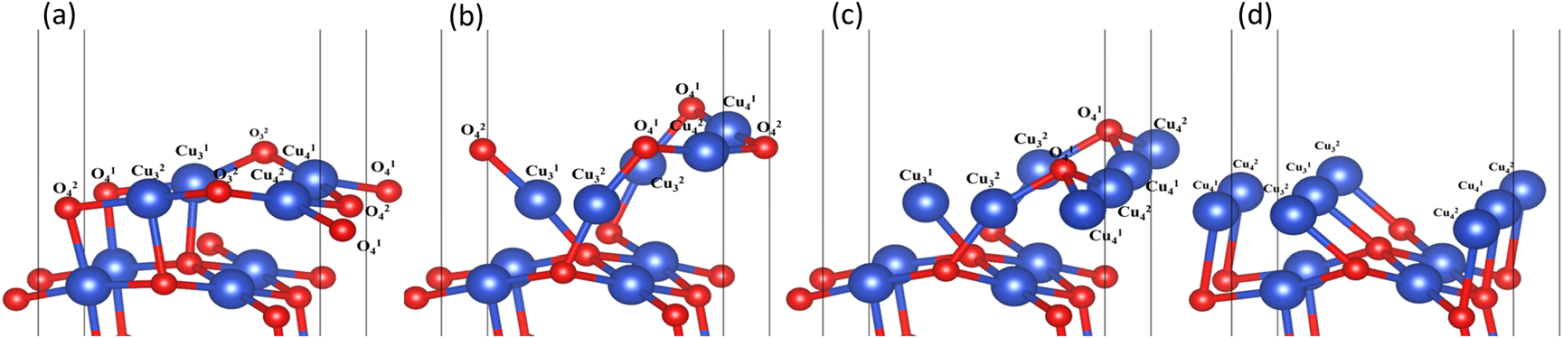
Surfaces with different oxygen vacancy concentrations as the reaction of H_2_ reduction of CuO occurs: (a) surface I (25% oxygen vacancy concentration), (b) surface II (50% oxygen vacancy concentration), (c) surface III (75% oxygen vacancy concentration), and (d) surface IV (100% oxygen vacancy concentration).

**Fig. 6:**
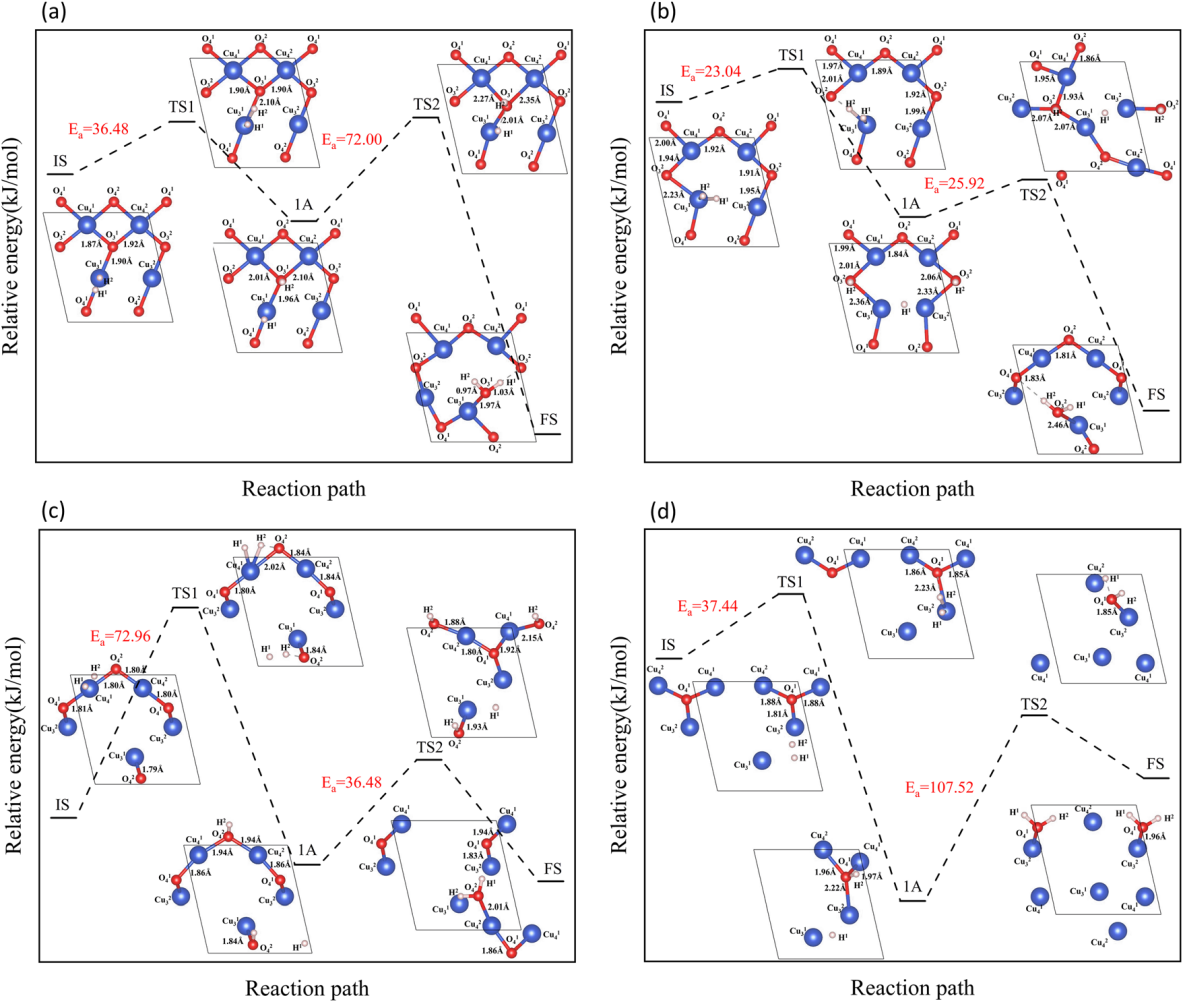
Entire process of the reaction path of H_2_ reduction on the CuO surface: (a) H_2_ reacts with an intact surface, (b) H_2_ reacts with surface I, (c) H_2_ reacts with surface II, (d) H_2_ reacts with surface III.

In the process of complete reduction of CuO by H_2_, as the reaction of H_2_ with surface O atoms proceeds, H_2_O is formed on the surface of CuO (111) with different oxygen vacancy concentrations until no O atoms remain on the surface. As shown in Fig. 5, for convenience, this article refers to the surfaces of CuO (111) with 25%, 50%, 75%, and 100% oxygen vacancy concentration as surface I, surface II, surface III, and surface IV, respectively. This designation was used to study the effect of different oxygen vacancy concentrations on the reaction between CuO and H_2_. The complete reaction process of H_2_ with the CuO (111) surface is shown in Fig. 6.

On the intact surface, the initial state was a state when H_2_ adsorbed on the top site of Cu^1^_3_, and the H–H bond length of the adsorbed H_2_ molecule was 0.75 Å, which was essentially unchanged compared to the H–H bond length of the gaseous H_2_ molecules (0.74 Å). Then, the H–H bond in the H_2_ molecule was broken to form an H–O group, and another H atom stayed in the top site of the Cu atom. During this process, the Cu–O bonds were activated, and the Cu–O bonds around O^1^_3_ lengthened. Subsequently, another H atom was combined with O^1^_3_ to form two H–O bonds on the surface. The two H–O bonds' lengths were 0.97 Å and 1.03 Å, respectively, and the H–O–H bond angle was 106.40°. In the H_2_O molecule, the H–O bond length is 0.99 Å, and the H–O–H bond angle is 104.5°. It can be considered that water molecules were generated on the surface.

On surface I, when H_2_ was adsorbed on the top site of Cu^1^_3_, the H–H bond length was 0.80 Å, and the H–H bond was activated. The Cu–O bond around O^2^_3_ was longer than that of the intact CuO surface, and when an H–O bond was formed, the Cu–O bonds were also more active than the ones on intact surfaces. Compared to the intact surface, on surface I, the energy barrier for the reaction between H_2_ and O^2^_3_ atoms to form H_2_O molecules was smaller, the reaction was more likely to occur, and more heat was given off by the entire reaction process. During the reaction, the O atoms on the surface were rearranged, with O^1^_4_ on the surface migrating to the original position of O^2^_3_. With the dissociation of H_2_O, a CuO surface with a 50% oxygen vacancy concentration, which has been denoted as surface II, was formed.

On surface II, the arrangement of Cu and O was similar to the (111) surface of Cu_2_O.^37^ The length of the Cu–O bond on this surface was shorter than that on both previous surfaces. At the initial state, H_2_ adsorbed on the top site of Cu, and the H–H bond length of the H_2_ molecule was 0.75 Å. They were not activated. The process of cleavage of H_2_ molecules and formation of an H–O bond had a higher energy barrier. The required energy to break the barrier for the subsequent formation of two H–O bonds was reduced but still high compared to the same reaction process on surface I.

On surface III, H_2_ cannot adsorb on the top sites of Cu, but instead adsorbed on the hollow sites on Cu atoms. The H–H bond length of the adsorbed H_2_ molecule was 0.75 Å. During the cleavage of the H_2_ molecule and the formation of an H–O bond, the required energy barrier was similar to the energy required for the first step of the reaction on the intact surface. The higher energy required in the formation of two H–O bonds may be due to the fact that more energy was required to rearrange the surface Cu atoms as more surface oxygen vacancies appeared.

In summary, the complete reduction of the CuO surface by H_2_ was calculated. The first chemical process in the two-step reaction was mainly the cleavage of the H_2_ molecule, the activation of the Cu–O bond, and the formation of the H–O bond. The second step was mainly the breaking of the Cu–O bond and the formation of the H–O–H bond. As the concentration of oxygen vacancies on the surface increased, the reaction between H_2_ and CuO was more intense on the surface with a 25% oxygen vacancy concentration. The reaction between H_2_ and CuO became more difficult and slowed down as the concentration of oxygen vacancies continued to increase.

### 3.2 Electronic structure analysis

Table 1 lists the Bader charge on the intact surface and on surfaces with four different oxygen vacancy concentrations (0%, 25%, 75%, and 100%), as well as the Bader charge of Cu atoms in CuO, Cu_2_O, and Cu bulks. According to the Bader charge results, it is concluded that on the intact surface, the three-coordinated Cu atoms carry more charge than the four-coordinated Cu atoms, which confirms the choice of three-coordinated Cu as the initial adsorption site for H_2_. The Bader charge of the O atom remained stable throughout the reaction. This was because the O atom is more electronegative with respect to the Cu atom and therefore always attracts charge to fill its empty orbitals. The Bader charge of the Cu atom increased as the reaction proceeded. Compared with the Bader charge of Cu atom in CuO bulk, on the intact surface, the four-coordinated Cu atoms were at +2 valence, and the three-coordinated Cu atoms were at +2 valence approximately because of the presence of surface dangling bonds. On surface II, the Bader charge of the Cu atoms were near the Bader charge of Cu atom in Cu_2_O bulk. Each Cu atom had two coordination bonds, which was consistent with the Cu atom in Cu_2_O bulk. It could be considered that the valence state of Cu atoms on surface II was +1. On surface IV, Cu atoms had no coordination bonds on the surface and only had one coordination bond with the O atoms on the subsurface. Comparing the Bader charge of Cu atoms on surface IV with the Bader charge of Cu atom in Cu bulk, it could be approximated that the Cu atoms on surface IV were at 0 valence. Therefore, during the reduction of a CuO surface by H_2_, the Cu atoms on the entire surface exhibited a change process of Cu^2+^ → Cu^1+^ → Cu^0^. Based on the calculation of the transition state, it was found that it is more difficult to reduce Cu^1+^ than Cu^2+^, a result that has also been confirmed by experiments.^9^

**Table 1:** Bader charge values of surface atoms under different oxygen vacancy concentration conditions and Cu atoms in CuO, Cu_2_O, and Cu bulks

Bader charge/*e*										
Surface	Atom									
	Cu^1^_3_	Cu^2^_3_	Cu^1^_4_	Cu^2^_4_	O^1^_3_	O^2^_3_	O^1^_4_	O^2^_4_	Bulk	Cu
Intact surface	10.24	10.24	10.02	10.02	6.90	6.90	6.91	6.91	CuO	10.00
Surface I	10.37	10.36	10.27	10.28		6.99	6.90	6.95		
Surface II	10.44	10.46	10.41	10.41			6.93	6.95	Cu_2_O	10.46
Surface III	10.73	10.50	10.71	10.67			6.92			
Surface IV	10.77	10.77	10.86	10.86					Cu	10.99

**Fig. 7:**
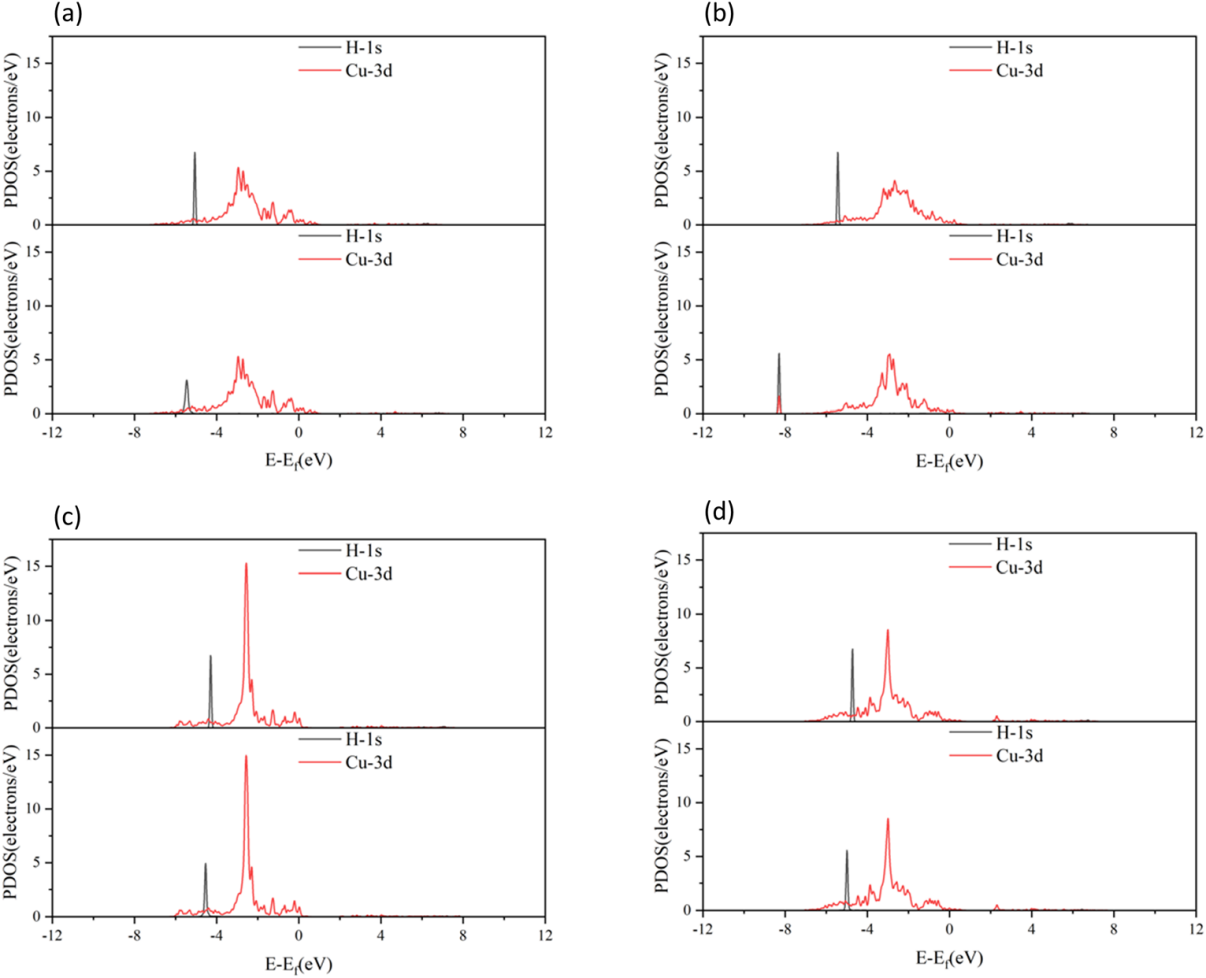
PDOS plots of H_2_ before and after adsorption on different CuO surfaces: (a) H_2_ adsorbed on the intact surface, (b) H_2_ adsorbed on surface I, (c) H_2_ adsorbed on surface II, and (d) H_2_ adsorbed on surface III. In all parts, the top figure shows before adsorption, and bottom figure shows after adsorption.

The molecular orbitals before H_2_ adsorption consisted of the σ_1s_ bonding orbital formed by the 1s orbitals of the two H atoms, and the 
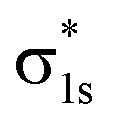
 antibonding orbital.^38^ The 
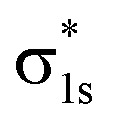
 antibonding orbitals were mainly located above the Fermi energy level, and with a small projected density of states (PDOS), so the electrons of H_2_ were mainly concentrated in the σ_1s_ bonding orbitals. The valence charge of the Cu atom was concentrated in the 3d orbital, which was the active orbital of the Cu atom.^39^Fig. 7 shows the PDOS plots of H 1s and Cu 3d before and after H_2_ adsorption on different surfaces. On the intact CuO surface, after H_2_ adsorption, the 1s orbitals of H were transferred to a lower energy level, whereas the 3d orbitals of Cu remain almost unchanged. The small overlap region between the two suggests that the hybridization between the 1s orbital of H and 3d orbital of Cu is weak, which corresponds to the relatively low adsorption energy. On surface I, the 1s orbitals of H and 3d orbitals of Cu after H adsorption generated new PDOS peaks at lower energy levels and overlapped. This indicated that, when H_2_ was adsorbed on surface I, hybrid bonding occurred between the 1s orbital of the H atom and the 3d orbital of Cu, forming a bonding orbital with lower energy, which increased the adsorption energy. At the same time, the original σ_1s_ and 
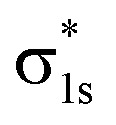
 molecular orbitals of H_2_ were destroyed, which led to the activation of the covalent bond of H_2_, which was conducive to the subsequent dissociation and diffusion of the H_2_. On surfaces II and III, after H adsorption, the H 1s orbital of moved to a lower energy level, the 3d orbital of Cu remained essentially unchanged, and the region of overlap between the two was relatively small. This indicated that on both surfaces the reaction with Cu remains weak when H_2_ molecular adsorption occurs. The analysis of the PDOS plots led to the conclusion that only surface I activated the covalent bond of H_2_ well upon adsorption of H_2_, which facilitated the subsequent dissociation of H_2_.

**Fig. 8:**
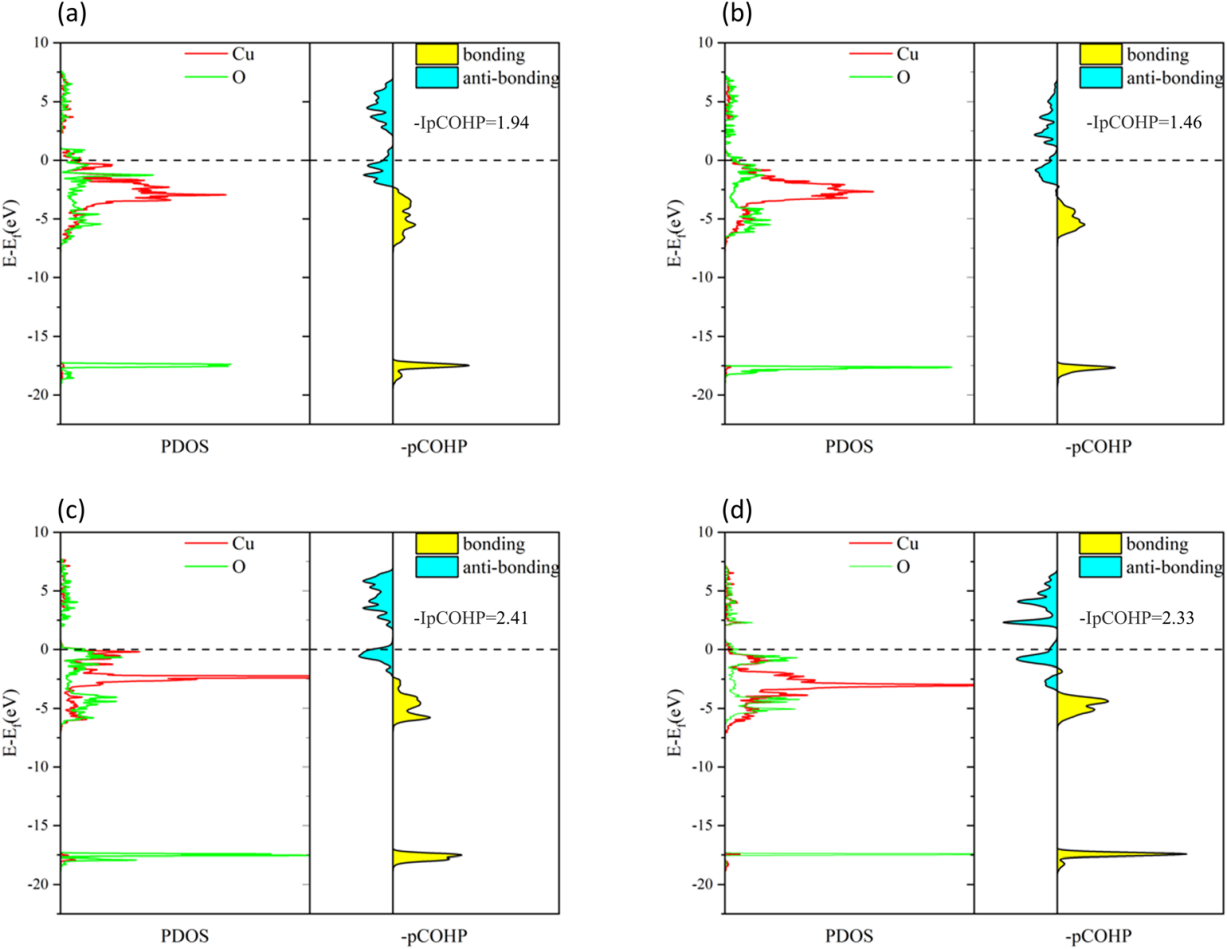
−pCOHP diagrams of Cu–O bonds on different CuO surfaces: (a) intact surface, (b) surface I, (c) surface II, and (d) surface III.

**Fig. 9:**
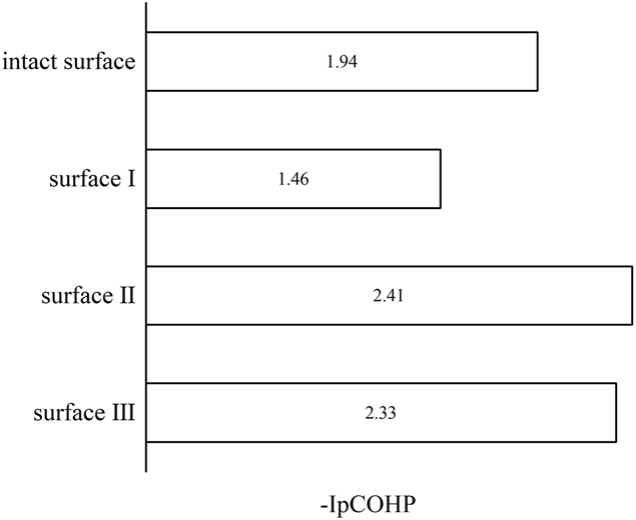
−IpCOHP diagrams of Cu–O bonds on different CuO surfaces.

To understand the bonding of Cu–O on different surfaces, Fig. 8 illustrates the minus projected crystal orbital Hamilton population (−pCOHP) diagrams of Cu–O bonds on CuO surfaces with different oxygen vacancy concentrations. The minus integrated projected crystal orbital Hamilton population (−IpCOHP) of the Cu–O bond is showed in Fig. 9. It can be found that the strength of the Cu–O bond decreased as the concentration of oxygen vacancies on the surface increased. However, as the oxygen vacancy concentration continued to increase, the surface structure underwent rearrangement to form the Cu_2_O phase. At this point, the strength of Cu–O bond increased again, and then decreased again with further increase in oxygen vacancy concentration. The strength of the Cu–O bond on surface I was reduced, which makes the reaction between H_2_ and the CuO surface more likely to occur. Less energy was needed to activate the Cu–O bond, and the entire reaction process occurred more easily. However, with the rearrangement of the surface structure, the strength of the Cu–O bond increased, making the reaction of H_2_ with surfaces II and III more difficult again, because the energy requirement for activating the Cu–O bond became higher.

Based on the analysis of the electronic structure, it can be concluded that an appropriate concentration of surface oxygen vacancies enhances the activation capacity of the CuO surface for H_2_ molecules and also reduces the strength of the Cu–O bond, which promotes the reaction of H_2_ with the surface O atoms. However, an excessive concentration of oxygen vacancies would cause rearrangements on the CuO surface, and the Cu_2_O phase formed would reduce the activation capacity for H_2_ molecules and enhance the strength of the Cu–O bond. At the same time, during the reduction of CuO by H_2_, excess oxygen vacancies caused a drastic rearrangement of Cu atoms on the surface of CuO, which increased the reaction energy barrier.

### 3.3 Subsurface oxygen transfer

The process of CuO reduction by H_2_ is not a single process in which H_2_ reacts with surface O atoms, but also involves the movement of subsurface O atoms to the surface. When oxygen vacancies appear on the surface, subsurface O atoms move upward. H_2_ reduction of the CuO surface consumes surface O atoms, and the upward movement of O from the subsurface replenishes the surface O atoms. The two processes compete with each other.

The CuO (111) model utilized in this study with zero H_2_ coverage is capable of simulating the transfer of subsurface O atoms under very low H_2_ concentration. As shown in Fig. 2, in the CuO (111) model, there are a total of two types of subsurface O atoms: one is the upward-connected, four-coordinated O^up^_sub_ and the other is the downward-connected, four-coordinated O^down^_sub_.

**Fig. 10:**
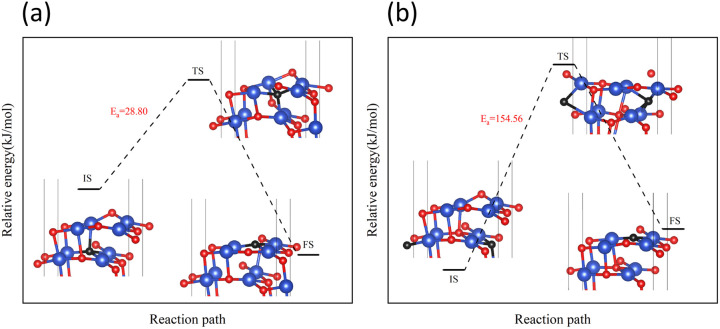
Transfer paths of two O atoms from the subsurface to the surface: (a) O^up^_sub_ transfers (b) O^down^_sub_ transfers.

On surface I, both subsurface O atoms could be transferred to the surface to replenish the O, returning the surface to an intact surface. The energy barriers for the upward transfer of these two O atoms were calculated separately. As shown in Fig. 10, on surface I, for the subsurface O atoms to move to the surface, they must first break the Cu–O bonds formed with the subsurface Cu atoms, reaching the transition state in the process. Subsequently, the O atom was bonded with the Cu atoms of the surface. The energy barrier for upward transfer of O^up^_sub_ was lower than that of O^down^_sub_, and the transfer of O^up^_sub_ to the surface was easier. However, the energy barrier for upward transfer of O atoms in both cases was higher than that for the reaction of H_2_ with O atoms on surface I (Fig. 6(b)). Therefore, for surface I, the rate of upward O replenishment at the subsurface was lower than the rate of surface O consumption by H_2_, and the CuO surface became surface II.

**Fig. 11:**
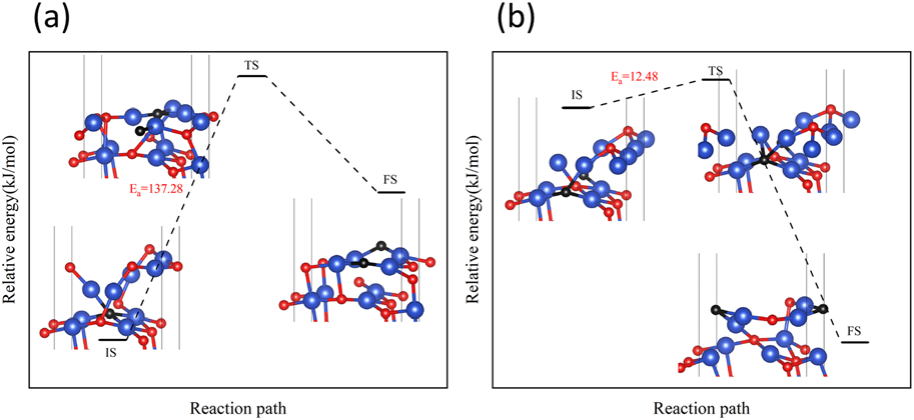
Transfer path of subsurface O^up^_sub_ to the surface on (a) surface II, and (b) surface III.

Next, the focus was the upward transfer of the subsurface O^up^_sub_, as shown in Fig. 11. For surface II, O^up^_sub_ transferred upward, returning the surface to surface I. The energy barrier is as shown in Fig. 11(a). It can be seen that the energy barrier is very high, and it is difficult for subsurface O to transfer upward. Therefore, the CuO surface changed from surface II to surface III.

However, for surface III, the upward transfer of subsurface O became quite easy, and had the upper hand in the competition with H_2_ consumption of O on the surface. The CuO surface returned to surface II; that is, the Cu_2_O phase on the surface would remain for a period of time. H_2_ continuously consumed O atoms on the surface, and subsurface O atoms were continuously transferred to the surface to replenish it, reaching a dynamic equilibrium. This coincides with the experimental observation of the reaction process Cu^2+^ → Cu^1+^ → Cu^0^ under low H_2_ concentration.

**Fig. 12:**
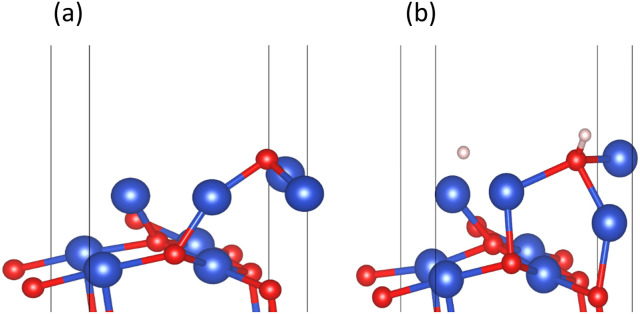
(a) Surface III and (b) surface III with 0.25 ML H_2_ coverage.

**Fig. 13:**
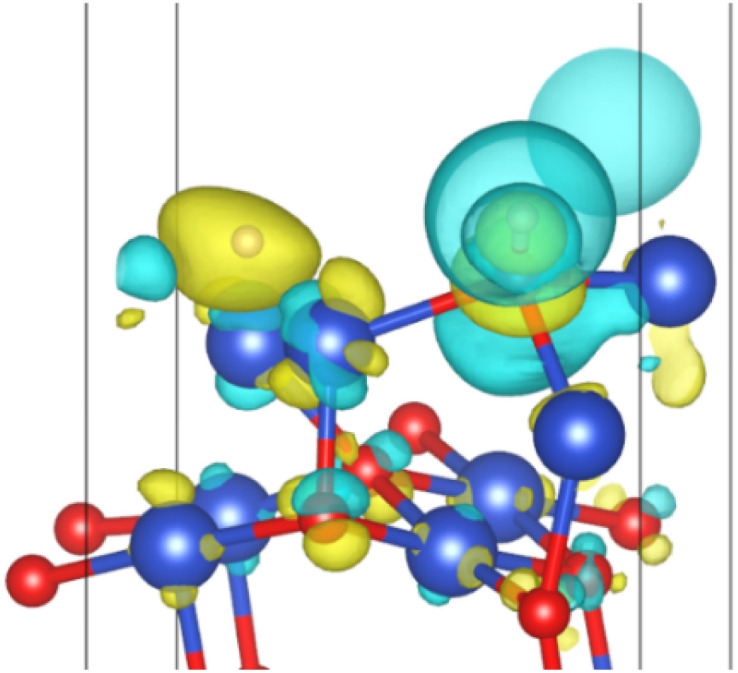
Surface charge transfer at 0.25 ML H_2_ coverage (yellow represents charge increase and blue represents charge decrease).

**Fig. 14:**
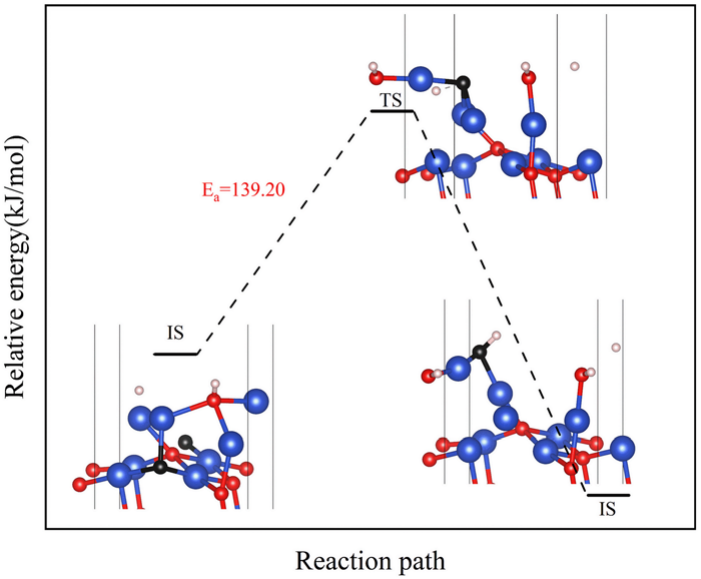
Transfer path of subsurface O^up^_sub_ to the surface at 0.25 ML H_2_ coverage.

However, in experiments, the Cu_2_O phase^7^ was not observed when the H_2_ concentration was high. The surface III with H_2_ coverage of 0.25 ML could simulate subsurface O atoms transfer under high H_2_ concentration, as shown in Fig. 12. This could be considered as having one H_2_ molecule on the surface III. However, this H_2_ molecule, after dissociation, occupied a surface oxygen vacancy and the top position of an O atom, respectively. Fig. 13 shows the charge transfer on the surface when there are H atoms on the surface. It can be seen that a large amount of charge was distributed around the H atoms on the oxygen vacancies. Fig. 14 illustrates the energy barrier for the O atom transfer process. For surface III, when oxygen vacancies were occupied by H atoms, the upward transfer of oxygen atoms required a large amount of energy to overcome the barrier. The transition state structure no longer occurred in the process of O atoms breaking the Cu–O bond with subsurface Cu atoms. Instead, it occurred in the process of forming H–O bonds between surface O and H. The transfer of subsurface O atoms was particularly difficult when the oxygen vacancies were occupied by H atoms. This may be due to the fact that H–O bonding replaces the original Cu–O bond breaking as a key step in the upward transfer of subsurface O. In the case of a high H_2_ concentration, surface III cannot return to surface II, which requires the replenishment of subsurface O. Instead, the surface changes to surface IV due to the reaction of the surface with H_2_. Therefore, when the H_2_ concentration is high, the Cu_2_O phase cannot be stabilized, and only the Cu^2+^ → Cu^0^ process can be observed in the experiment.

### 3.4 Thermodynamic analysis

The thermodynamic analysis of the H_2_ reduction process of CuO was investigated. According to relevant papers on thermodynamics, the change of Gibbs free energy (Δ*G*) can predict the direction and spontaneity of chemical reactions.^40^ Fe_3_O_4_, Co_3_O_4_ and NiO are other common oxygen carriers for chemical looping combustion.^41–43^ The thermodynamic analysis of the reaction between these other common oxygen carriers and H_2_ was used for comparison with the reaction between CuO and H_2_. The reactions occurring during the H_2_ reduction of CuO and the reactions of other common oxygen carriers with H_2_ are listed below.

5 H_2_ + 2CuO → H_2_O + Cu_2_O

6 H_2_ + Cu_2_O → H_2_O + 2Cu

7 H_2_ + CuO → H_2_O + Cu

8 H_2_ + 0.25Fe_3_O_4_ → H_2_O + 0.75Fe

9 H_2_ + 0.25Co_3_O_4_ → H_2_O + 0.75Co

10 H_2_ + NiO → H_2_O + Ni

**Fig. 15:**
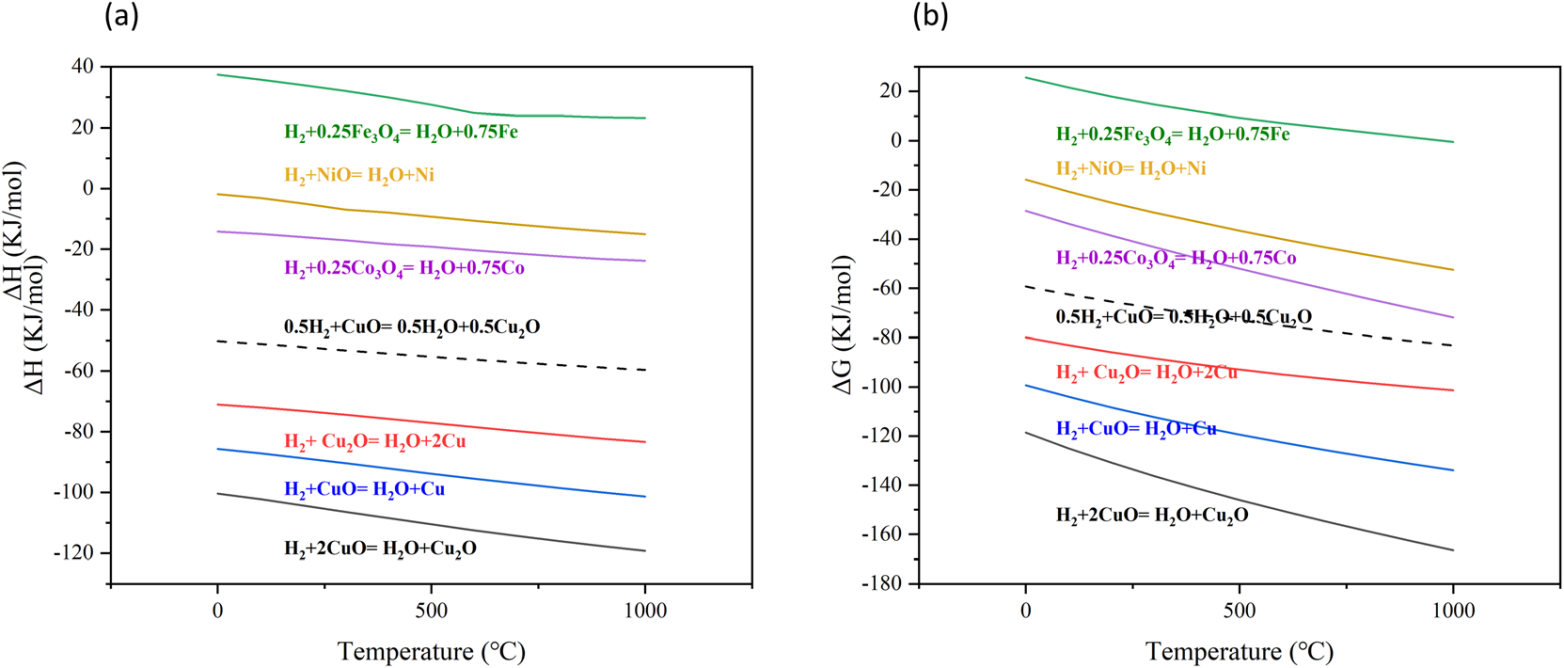
The trend of Δ*H* and Δ*G* for each reaction with temperature changes.

Δ*H* and Δ*G* of each reaction are shown in Fig. 15. All reactions (reaction (5)–(10)) show the same trend. As the temperature increased, both Δ*H* and Δ*G* decreased, indicating that the reactions were more favorable at higher temperatures. The reduction of CuO to Cu_2_O by H_2_ (reaction (5)) released more heat compared to the reduction of Cu_2_O to Cu by H_2_ (reaction (6)). According to the Δ*G*, it could be concluded that reducing CuO to Cu_2_O by H_2_ is easier than reducing Cu_2_O to Cu by H_2_. The DFT calculation in this study obtained the same result that the reduction of CuO to Cu_2_O by H_2_ was more likely to occur and released more heat as thermodynamic analysis. The reaction between Fe_2_O_3_ and H_2_ was an endothermic reaction (reaction (8)). The reaction between NiO and H_2_ was exothermic, but the heat released was small (reaction (10)). Compared to the reaction between NiO and H_2_, the reaction between Co_3_O_4_ and H_2_ released more heat (reaction (9)). However, in contrast to these common oxygen carriers, the reaction between CuO and H_2_ released much more heat. The molar ratio of H_2_ to CuO in reaction (5) was 1 : 2 and 1 : 1 in reaction (6). Compared to reaction (6), reaction (5) required more CuO at the same molar amount of H_2_. It can be observed that when the H_2_ concentration was low, the amount of CuO was sufficient, and reaction (5) was thermodynamically favorable. However, as the H_2_ concentration increased, the amount of CuO limited the reaction. Under the same amount of CuO, reaction (6) was more likely to occur.

## 4 Conclusions

In this study, DFT and thermodynamic calculation were used to study the whole process of the reaction between the CuO (111) surface and H_2_ as well as the transfer of subsurface O atoms at different H_2_ coverage (0 ML, 0.25 ML). Throughout the entire process of H_2_ reduction of the CuO (111) surface, the surface Cu atoms underwent the transformation process of Cu^2+^ → Cu^1+^ → Cu^0^. As the reaction proceeded, the concentration of surface oxygen vacancies increased, and the CuO surface underwent rearrangement of O atoms to form the Cu_2_O phase. The Cu–O bond of the Cu_2_O phase was more stable than the Cu–O bond of the CuO phase, and it was therefore more difficult to reduce Cu_2_O with H_2_ than to reduce CuO. The surface H_2_ coverage could reflect the concentration of H_2_. As the surface oxygen vacancy concentration increased, it was more likely that the subsurface O atoms transfer to the surface at zero H_2_ coverage, allowing the surface to maintain a stable Cu_2_O phase. However, when the H_2_ coverage was 0.25 ML, the H atoms on the surface hindered the upward transfer of O atoms from the subsurface; therefore, O atoms on the surface was not replenished timely, and the Cu_2_O phase could not be maintained stably. Through thermodynamic analysis, compared to other oxygen carriers (Co_3_O_4_, NiO), the reaction between CuO and H_2_ released more heat. The concentration of H_2_ affects the reaction products of H_2_ with CuO. In summary, the concentration of H_2_ affected the valence state of surface Cu by affecting the transfer of subsurface O atoms. It could be considered that as long as the surface oxygen vacancies were occupied by H atoms, subsurface O atoms could not transfer upward in a timely manner, preventing the stable existence of the Cu_2_O phase. However, there are various arrangements of H atoms on the surface of CuO, and this study only investigated one particular configuration to examine its impact on subsurface O atom transfer. The study of the effects of different arrangements of H atoms on the surface of CuO will be a primary focus for future research. Meanwhile, AIMD and KMC are two other excellent methods for studying chemical reactions. The application of AIMD and KMC methods will also become the focus of our future work.

## Author contributions

Y. Wu performed the calculations and the data analysis and R. Fang prepared the draft of the manuscript. L. Shen initialized the project and proposed the supervision. H. Bai provided the VASP software copyright. All authors have given approval to the final version of the manuscript.

## Conflicts of interest

There are no conflicts to declare.

## Supplementary Material
